# Supplementary material for: A secondary RET mutation in the activation loop conferring resistance to vandetanib
Source: Nat Commun. 2018 Feb 12;9:625. doi: 10.1038/s41467-018-02994-7 (PMC5809600; doi:10.1038/s41467-018-02994-7)
Supplement: Supplementary file 1 — Supplementary Information [file 41467_2018_2994_MOESM1_ESM.pdf]

Supplementary Information

A Secondary *RET* Mutation in the Activation Loop Conferring Resistance to Vandetanib

TABLE OF CONTENTS

Supplementary Figures 1–9..3

Supplementary Tables 1-2...22

References...24

Takashi Nakaoku[1], Takashi Kohno[1,2], Mitsugu Araki[3,4], Seiji Niho[5], Rakhee Chauhan[6], Phillip P. Knowles[6], Katsuya Tsuchihara[2], Shingo Matsumoto[2,5], Yoko Shimada[1], Sachiyo Mimaki[2], Genichiro Ishii[7], Hitoshi Ichikawa[2], Satoru Nagatoishi[8], Kouhei Tsumoto[8], Yasushi Okuno[4], Kiyotaka Yoh[5], Neil Q. McDonald[6,9], Koichi Goto[5]

[1]Division of Genome Biology, National Cancer Center Research Institute, 5-1-1, Tsukiji, Chuo-ku, Tokyo, 1040045, Japan.

[2]Division of Translational Genomics and [7]Division of Pathology, Exploratory Oncology Research and Clinical Trial Center, National Cancer Center, 5-1-1, Tsukiji, Chuo-ku Tokyo, 1040045 and 6-5-1, Kashiwanoha, Kashiwa-City, Chiba, 2778577, Japan.

[3]Advanced Institute for Computational Science, RIKEN, 7-1-26 Minatojima-minami-machi, Chuo-ku, Kobe-city, Hyogo, 6500047, Japan.

[4]Department of Clinical System Onco-Informatics, Graduate School of Medicine, Kyoto University, 54 Kawaracho, Shogoin, Kyoto-city, Kyoto, 6068507, Japan.

[5]Department of Thoracic Oncology, National Cancer Center Hospital East, 6-5-1, Kashiwanoha, Kashiwa-city, Chiba, 2778577, Japan.

[6]Signaling and Structural Biology Laboratory, The Francis Crick Institute, London, NW1 1AT, UK.

[8]Medical Proteomics Laboratory, Institute of Medical Science, The University of Tokyo, 4-6-1, Shiroganedai, Minato-ku, Tokyo, 1088639, Japan.

[9]Institute of Structural and Molecular Biology, Department of Biological Sciences, Birkbeck College, Malet Street, London WC1E 7HX, UK.

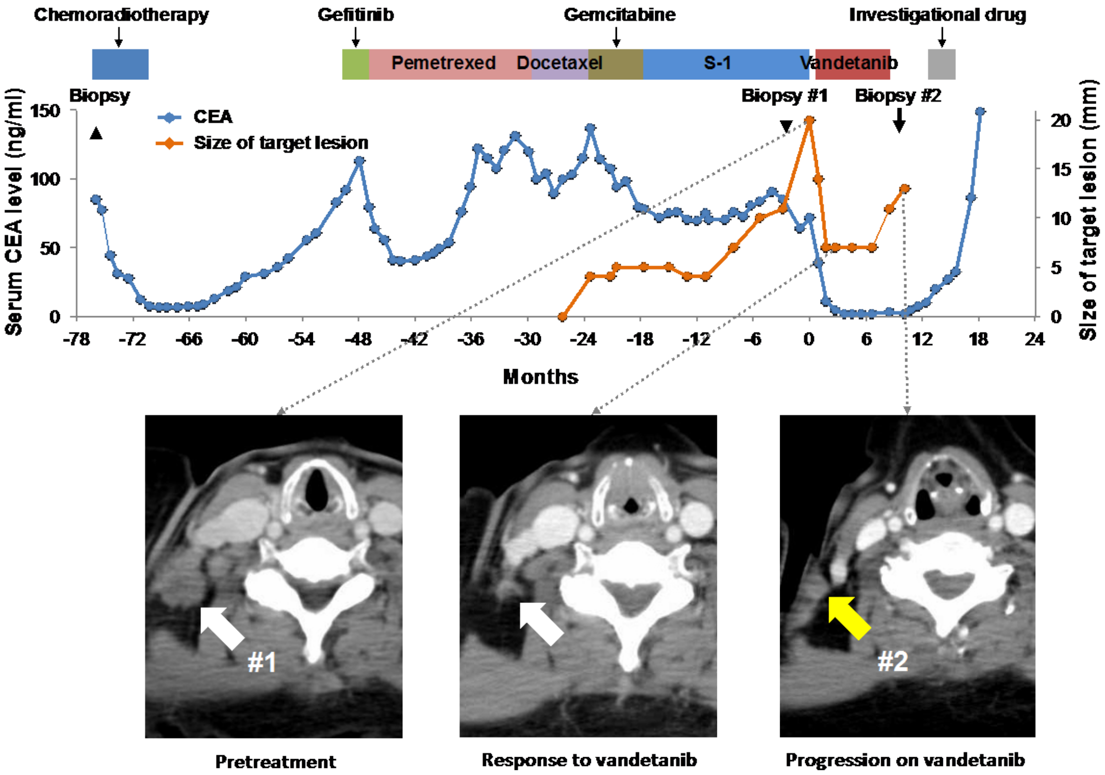

Supplementary Figure 1. Complete clinical course of the patient

A 57-year-old Japanese woman was referred to our hospital for assessment of a nodule in her left lung that was detected in a medical checkup. Bronchoscopic and mediastinoscopic examinations revealed adenocarcinoma of the lung with mediastinal lymph node metastases. The patient underwent concurrent chemoradiotherapy with cisplatin and vinorelbine, resulting in a partial response; however, multiple bone metastases were detected 2 years later. Genetic examination revealed no mutation in *EGFR*. The patient received second- to sixth-line chemotherapies consisting of gefitinib, pemetrexed, docetaxel, gemcitabine, and S-1. During sixth-line chemotherapy, the patient developed right cervical lymphadenopathy, and biopsy of the lymph node

again revealed adenocarcinoma (*bottom* left, indicated as #1 by a white arrow). A *CCDC6-RET* fusion was detected by reverse transcriptase–polymerase chain reaction (RT-PCR) analysis of total RNA extracted from the snap-frozen biopsied tumor cells in a nation-wide genetic screen, LC-SCRUM-Japan (Lung Cancer Genomic Screening Project for Individualized Medicine in Japan). The patient was enrolled into our investigator-initiated clinical trial, LURET (Lung Cancer with RET Rearrangement Study; clinical trial registration number: UMIN000010095), and began treatment with vandetanib (300 mg, once daily). A computed tomographic (CT) scan of the chest obtained after 12 weeks showed a decrease in the short axis of the metastatic cervical lymph node from 20 to 7 mm (65% reduction) in response to vandetanib treatment (*bottom* middle). However, another cervical lymphadenopathy developed 38 weeks after initiation of treatment (*bottom* right, indicated as #2 by a yellow arrow). A biopsy of the newly developed lymph node was performed. The blue line indicates the serum CEA level, and the orange line indicates the size of the target lesion (the right metastatic cervical lymph node).

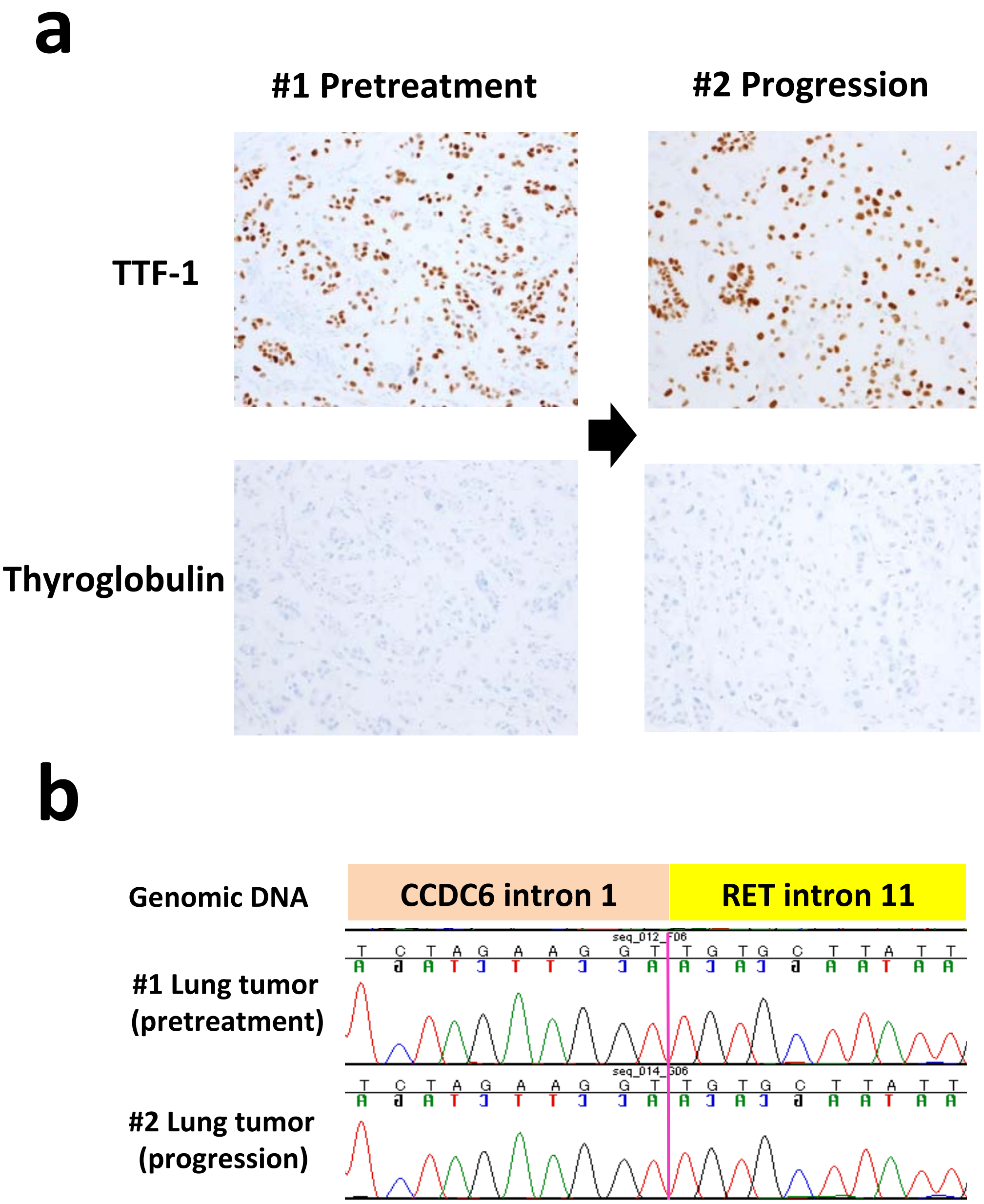

Supplementary Figure 2. Pathological and genomic features of the recurrent tumor

(a) Lymph node biopsy specimens were stained for two biomarkers for the diagnosis of lung adenocarcinoma, TTF-1 and thyroglobulin, before treatment (*left*, biopsy #1) and at the time of progression (*right*, biopsy #2).

(b) Sanger sequencing of genomic-PCR products encompassing the breakpoint junction. Genomic DNAs from the two biopsy specimens obtained before treatment and at the time of progression showed the same breakpoint junction, confirming the presence of the same *CCDC6-RET* fusion in both specimens. Given the high diversity of breakpoints for *RET* fusions[1], this result confirmed that the resistant tumor originated from the tumor present before vandetanib treatment.

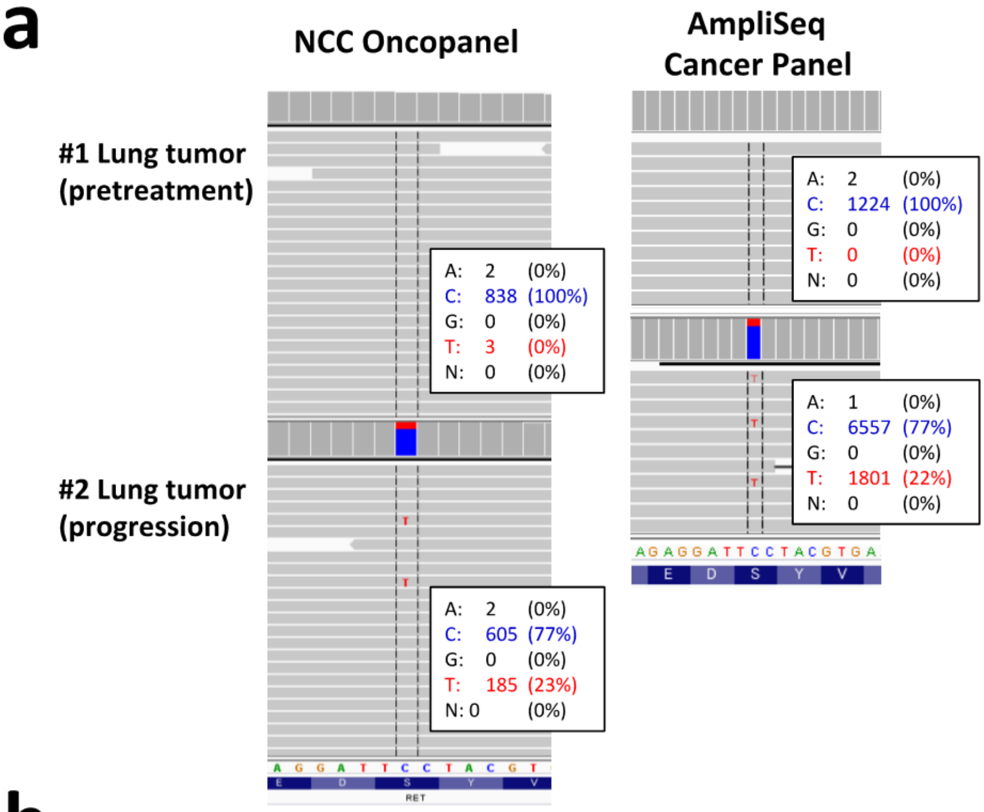

b

Sample	Estimated tumor cellularity (%)		Target sequencing	Exome sequencing			
		Gene	*RET*	*PPP1R9A*	*RET*	*SP3*	*SYNE2*
		Chromosome	chr10	chr7	chr10	chr2	chr14
		Position	43,615,632	94,827,698	43,615,632	174,820,338	64,599,018
		Reference/variant	C/T	G/A	C/T	T/C	G/C
		AA change	S904F	E598K	S904F	Q301R	L4792F
			Variant allele fraction (no. of variant reads/total reads)				
Blood	0		Not tested	0 (0/63)	0 (0/14)	0 (0/94)	0 (0/16)
Lung tumor (Pretreatment)	42		0 (3/843)	0 (0/506)	0 (0/93)	0 (0/513)	0 (0/56)
Lung tumor (Progression)	75		23 (185/792)	13.1 (51/388)	20.7 (12/58)	29.7 (94/316)	26.4 (14/53)

Supplementary Figure 3. Identification of an acquired RET mutation

(a) Genome images from the Integrative Genomics Viewer (Broad Institute) for the targeted deep-sequencing analyses of genomic DNA, revealing a mutation

of cytosine 2,902 to thymine (serine 904 to phenylalanine) observed only in progressive disease. (*Left*) Results of the analysis performed using a NCC Oncopanel to examine 90 cancer-related genes. (*Right*) Results of the analysis performed using an Ion AmpliSeq Cancer Hotspot Panel v2 (Thermo Fisher Scientific) covering 52 cancer-related genes.

(b) Next-generation sequencing of tumor samples before treatment and at progression. The results of targeted deep-sequencing analyses and whole-exome sequencing analyses are shown. Whole-exome sequencing revealed four mutations specific to the recurrent tumor, including the *RET*-S904F mutation.

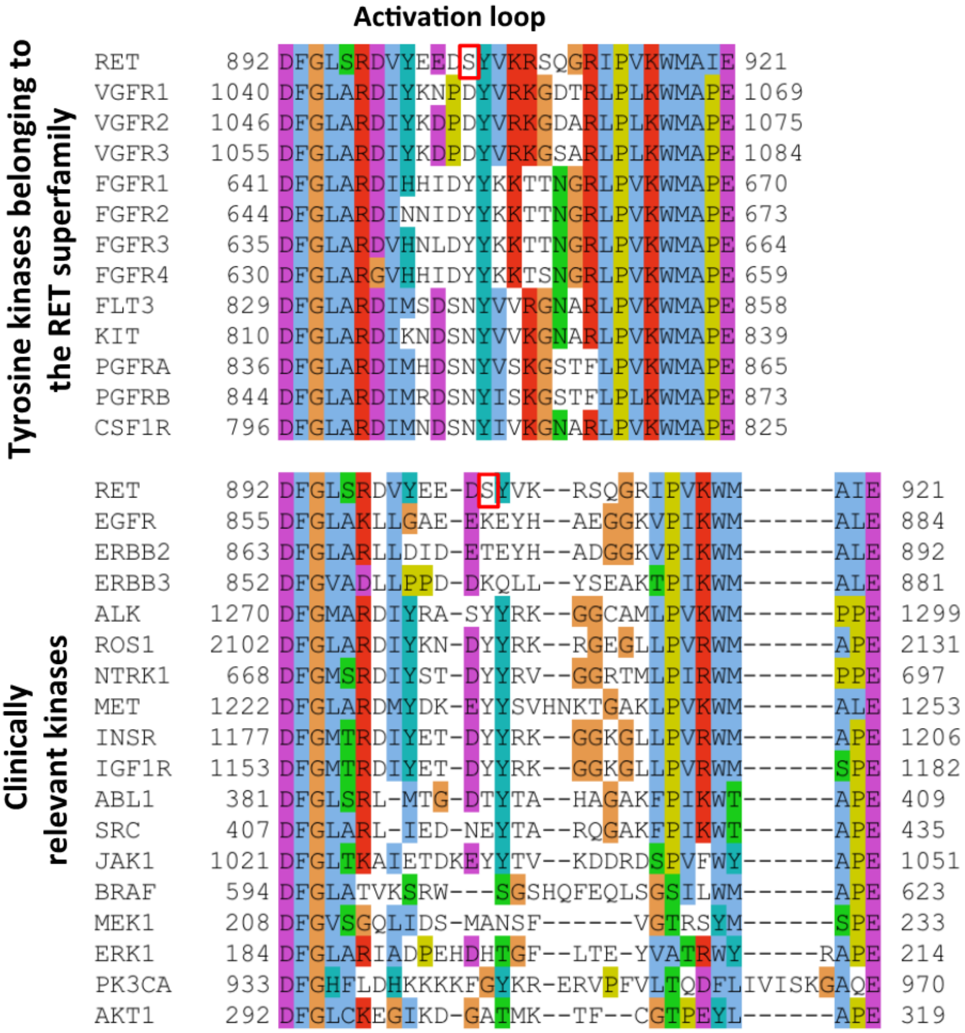

Supplementary Figure 4. Alignments of the amino acid sequences of the activation loop of RET and other kinases

The serine residue at position 904 of RET is located in the activation loop of the catalytic domain (residues 900–905). The S904 residue is not conserved among human tyrosine kinases belonging to the RET superfamily or clinically relevant tyrosine and serine–threonine kinases. Tyrosine kinases belonging to the RET superfamily were listed via the Ensembl Genome Browser (www.ensembl.org) and the human kinome[2], whereas other kinases were selected based on their clinical utility as drug targets.

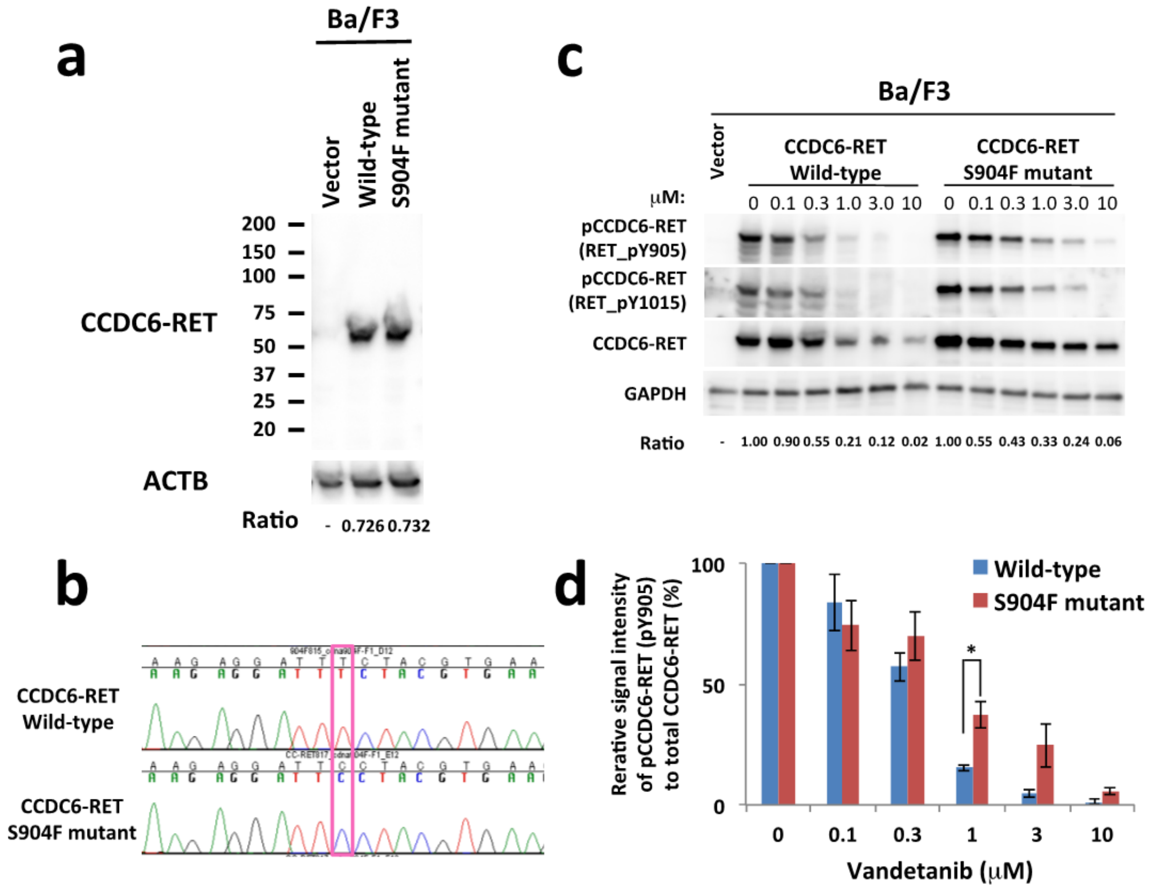

Supplementary Figure 5. Resistance of the S904F mutant CCDC6-RET fusion protein to vandetanib in Ba/F3 cells

(a) Immunoblot analysis showing the predicted size of CCDC6-RET fusion proteins (56 kDa) from IL3-independent Ba/F3 cells stably expressing the full-length wild-type and S904F mutant CCDC6-RET proteins. CCDC6-RET protein expression was similar in the two cells. The ratio was calculated by dividing the signal intensity of the CCDC6-RET protein by that of ACTB after subtraction of the background.

(b) Sanger sequencing of genomic products from Ba/F3 cells, confirming a

mutation of cytosine to thymine at residue 2,902 in the S904F mutant.

(c) Immunoblot analysis of Ba/F3 cells stably expressing the wild-type or S904F mutant CCDC6-RET fusion protein. Cell lysates were extracted at 6 h after exposure to 0, 0.1, 0.3, 1.0, 3.0, or 10 μM vandetanib.

(d) The mean ratios of phospho-Ret (pTyr905) to total RET from three separate experiments are shown with standard deviation from the experiment in (**c**) performed separately three times. The graph shows the mean ratios of phospho-Ret (pTyr905) to total RET from three separate experiments with standard deviations (shown as error bars). Concentrations showing statistical significance ($p < 0.05$ by t-test) are marked with an asterisk. ACTB: beta-actin.

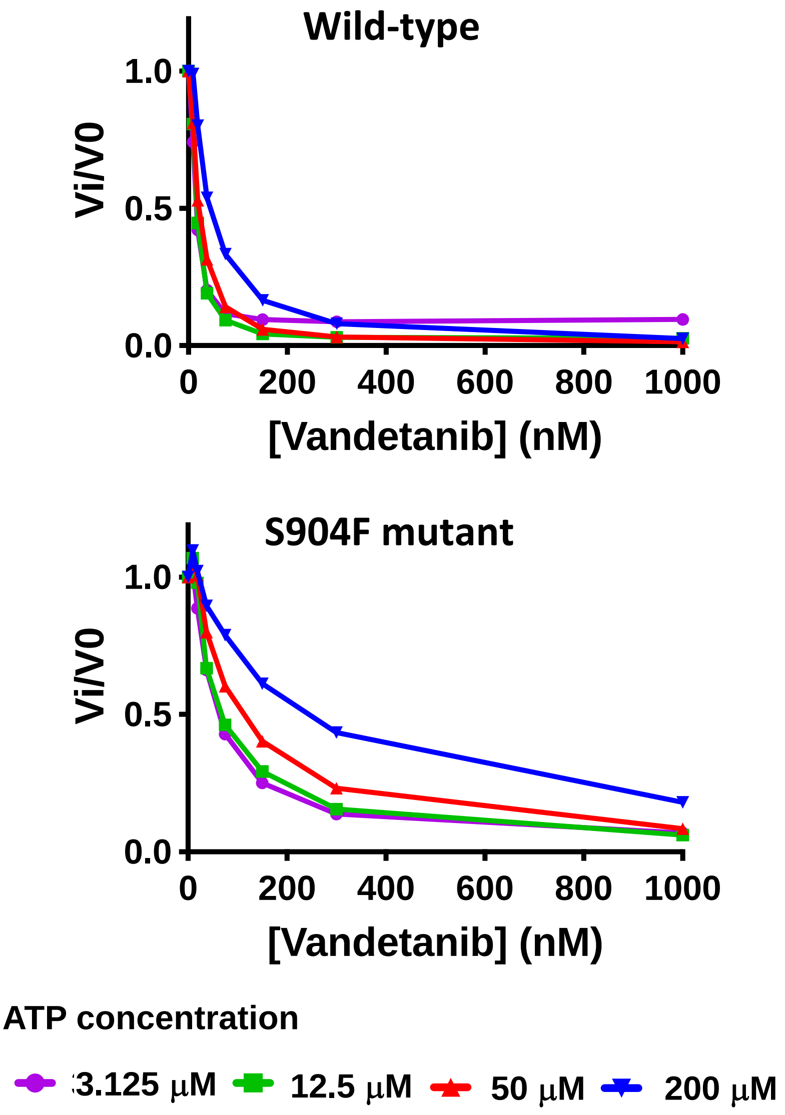

Supplementary Figure 6. Kinetic enzyme assay using purified RET kinase domain polypeptides

Inhibited velocities (Vi) of wild-type (*upper*) and S904F mutant (*lower*) RET KDs treated with vandetanib in the presence of ATP (3.125, 12.5, 50.0, and 200 nM). Reactions were performed in triplicate in a reaction mixture containing serially diluted vandetanib. The graph shows the ratio of the inhibited to control *in vitro* kinase activities of the RET KDs with or without the S904F mutation measured

in the presence of serially diluted vandetanib using the IGF1Rtide synthetic peptide substrate (KKKSPGEYVNIEFG). An increase in ATP concentration caused a decrease in the inhibitory effects of vandetanib, an ATP-competitive TKI, as predicted. The inhibitory constants were calculated by fitting to the non-linear regression method using GraphPad Prism version 6.0 (**Figure 3a**).

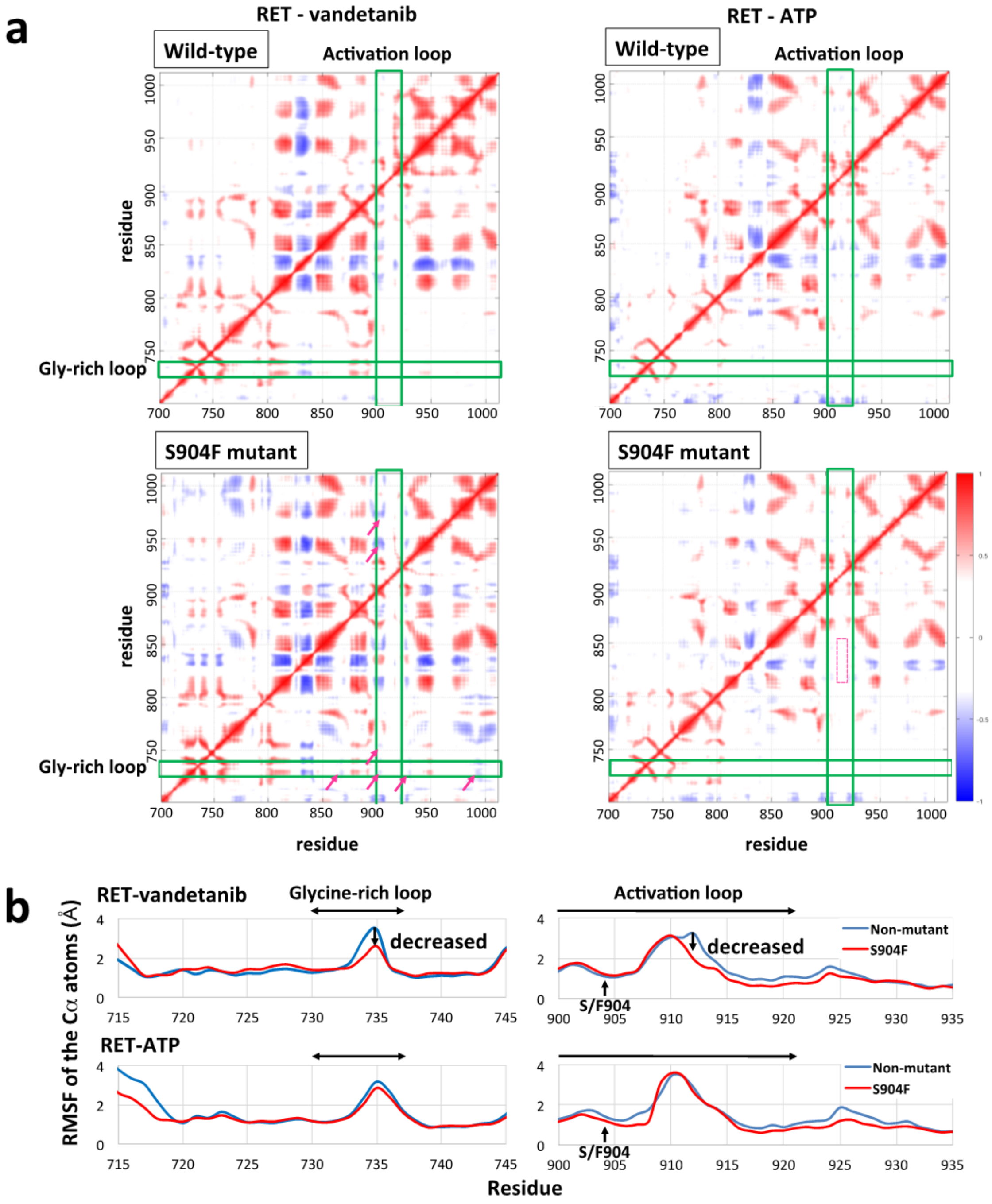

a
RET - vandetanib
RET - ATP
Wild-type
Activation loop
S904F mutant
Gly-rich loop
residue
b
RET-vandetanib
RET-ATP
Glycine-rich loop
Activation loop
decreased
Non-mutant
S904F
S/F904
RMSF of the Cα atoms (Å)
Residue

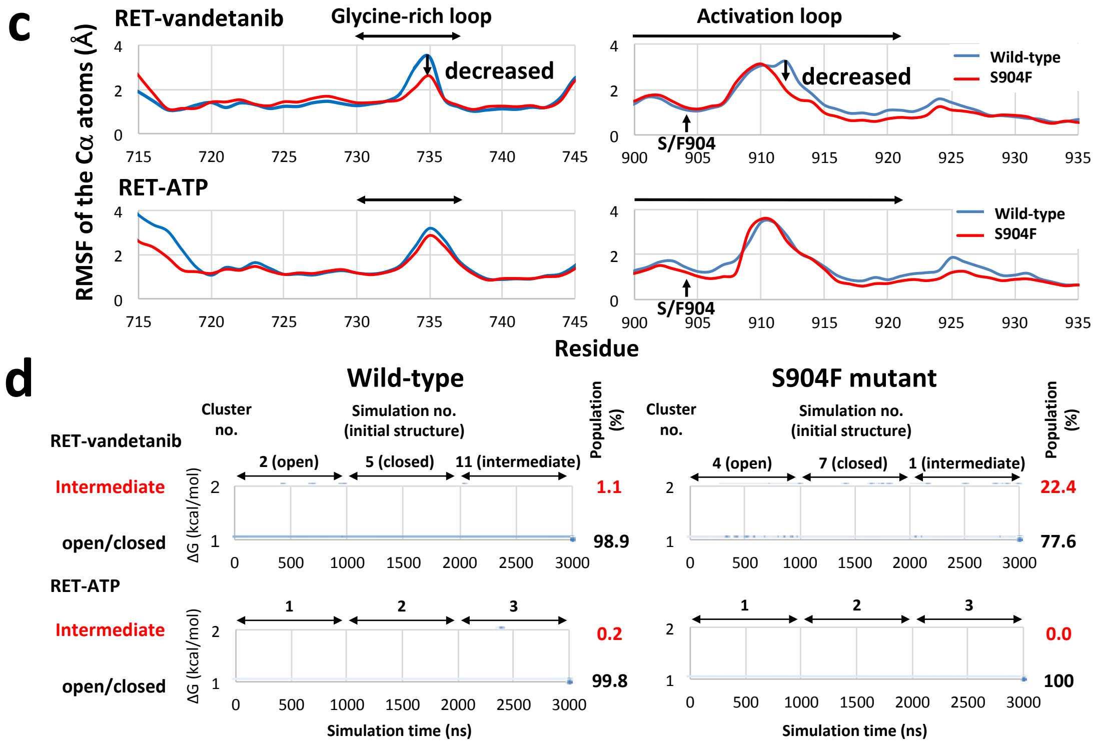

e

RET kinase	Conformer	Complex: Vandetanib	Complex: ATP
		⊿G (kcal/mol) Population (%)	⊿G (kcal/mol) Population (%)
Wild-type	Open/closed	−13.07 ±0.22 (98.9)	−11.8 ±1.4 (99.8)
	Intermediate	not calculated* (1.1)	not calculated (0.2)
S904F mutant	Open/closed	−14.28 ±1.12 (77.6)	−12.8 ±0.8 (100)
	Intermediate	−11.16 ±0.64 (22.4)	not calculated (0)

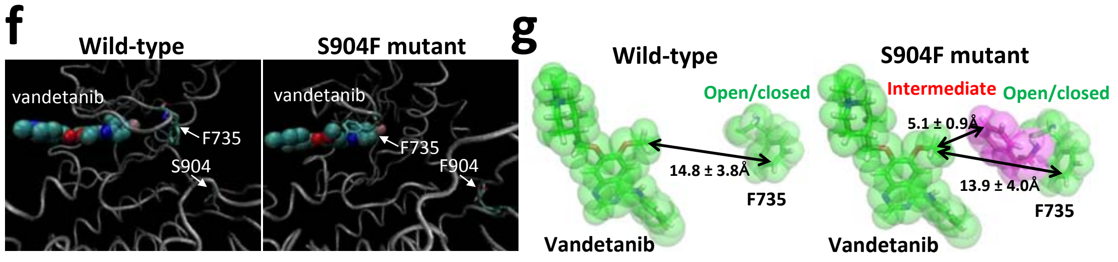

Supplementary Figure 7. Molecular dynamics (MD) of RET kinase in complex with vandetanib

(a) The dynamic cross-correlations in wild-type/mutant RET bound to vandetanib/ATP were analyzed using molecular dynamics trajectories of 1 μs × 3. After the backbone Cα atoms in RET were structurally aligned to remove

rotational and translational motion, the dynamic cross-correlation coefficient between each Cα atom was calculated as described previously[3,4]. Compared with the map of non-mutant RET, the emergence and disappearance of dynamic correlations involving GRL and AL are indicated by *arrows* and a *dashed box*, respectively. In the presence of vandetanib, but not of ATP, novel dynamic coupling between GRL and AL was evident in the S904F mutant KD.

(b) Root-mean-square fluctuation (RMSF) of backbone Cα atoms for residues 715–745 (*left*) and 900–935 (*right*) in RET in complex with vandetanib and ATP. RMSF values were calculated using MD trajectories of 1 μs × 3. In the presence of vandetanib, but not of ATP, the S904F mutation decreased the flexibility of proximal residues 910–915 in the activation loop (AL), concomitantly with that of distant residues 733–735 in the glycine-rich loop (GRL) (locations are indicated by arrows). This change was thought to result in the distortion of the hydrogen-bond network formed by E734 in the GRL, and D771 and R912 in the AL (see also, **Figure 3b**).

(c) Dynamic structures of the S904 wild-type (*left*) and F904 mutant (*right*) RET in complex with vandetanib. Two nanosecond mean structures observed in simulations of 50 ns × 15 times were superimposed. F735, S/F904, and vandetanib are indicated. F735 in the *open/closed* and *intermediate* conformations is represented by green and magenta sticks, respectively, based on clustering of the 375 2 ns mean structures. The population of the *intermediate* conformation was evident only in the F904 mutant (*right*). Green/magenta, carbon; blue,

nitrogen; orange, phosphorus; red, oxygen; light blue, fluorine; and light pink, bromine.

(d) Conformational states of the glycine-rich loop (Gly731–Lys737) of wild-type and S904F mutant RET KDs in complex with vandetanib (*upper*) and ATP (*lower*) as determined by simulations. Two nanosecond mean structures extracted from simulations (1 μs × 3, 1,500 structures) were hierarchically clustered as described in "Clustering of MD structures of the RET-vandetanib complex" in Methods. The data show two energetically distinct conformers of vandetanib binding, where Cluster nos. 1 and 2 correspond to the *open/closed* and *intermediate* conformers, respectively. The percentage of each conformational population was calculated for wild-type (*left*) and S904F mutant (*right*) KDs, indicating that the *intermediate* conformer occurred preferentially in simulations of the S904F mutant KD in complex with vandetanib (*upper*). A percentage of the population of the energetically clustered structure is shown on the right of each graph.

(e) Conformational states and binding free energies (ΔG) of RET kinase in complex with vandetanib and ATP. The binding free energy values (ΔG) were calculated by MP-CAFEE. A population of RET kinase in complex with vandetanib in each conformational state was calculated using long-time (1 μs) MD trajectories. (*) ΔG for wild type–vandetanib in intermediate conformation was not calculated because the conformation was observed too transiently in both short and long MD trajectories, indicating that the conformation is

unstable for wild-type RET in complex with vandetanib.

(f) Snapshots of Supplementary Movies 1 (*left*) and 2 (*right*) representing 1 μs MD simulations of the human RET kinase domain in complex with vandetanib. Each movie represents a 1 μs simulation for wild-type RET (simulation no. 5) (*left*) and S904F RET mutant (simulation no. 4) (*right*) KDs in (**d**). F735 and S/F904 are represented by thick sticks, and vandetanib is represented by Corey–Pauling–Koltun space-filling models. Green, carbon/fluorine; blue, nitrogen; red, oxygen; and light pink, bromine.

(g) Deduced interactions between vandetanib and F735 in the S904 wild type (*left*) and F904 mutant (*right*). The mean structures of F735 and vandetanib generated by simulations of 50 ns × 15 are represented by thick stick and Corey–Pauling–Koltun space-filling models[5]. The mean structure of the *open*/*closed* conformers clustered in a low free-energy state and that of the *intermediate* conformer clustered in a high free-energy state (**e**) are depicted in green and magenta, respectively. *Open* and *closed* conformers were energetically equivalent and aggregated into a low free-energy state. All structures observed in the 15 trajectories were used to calculate the atomic distance between Cζ in F735 and the methyl carbon in vandetanib as 14.8 ± 3.8, 13.9 ± 4.0, and 5.1 ± 0.9 Å for the wild type in the *open*/*closed* conformer, S904F mutant in the *open*/*closed* conformer, and S904F mutant in the *intermediate* conformer, respectively.

Figure 2a (Original immunoblots)

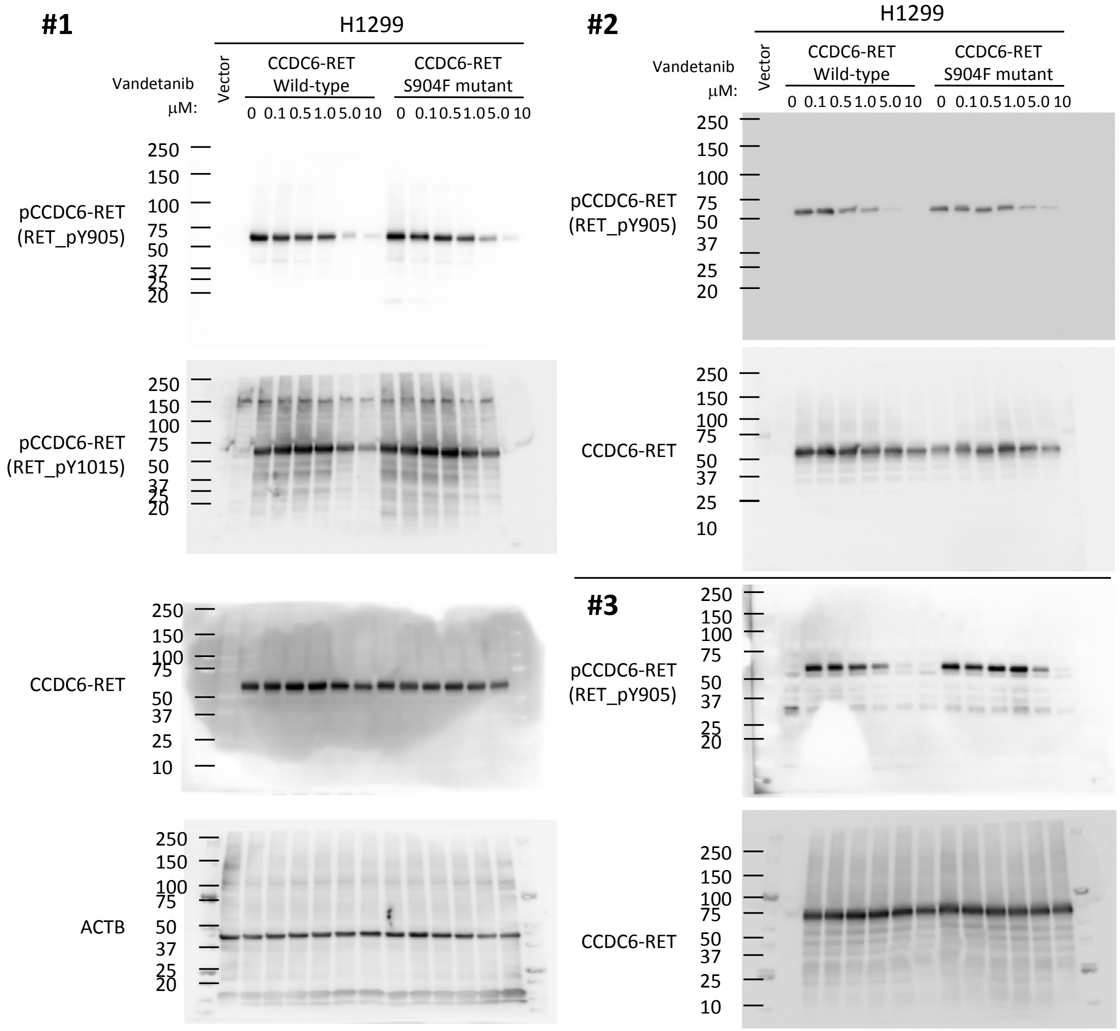

Supplementary Figure 8. Sources of immunoblotting data

The experiments were independently repeated three times. **Figure 2a** was composed of data #1. The graph of **Figure 2a** was calculated using all three experiments.

Supplementary Figure 9

pY

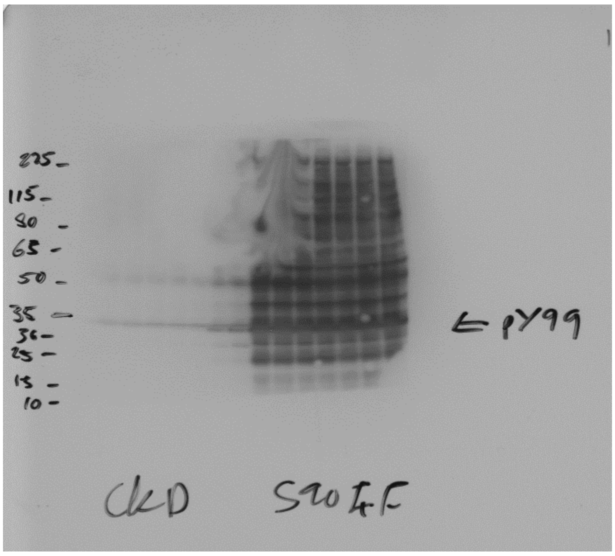

pY905

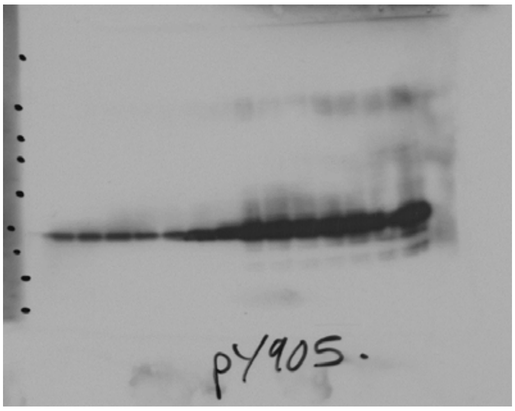

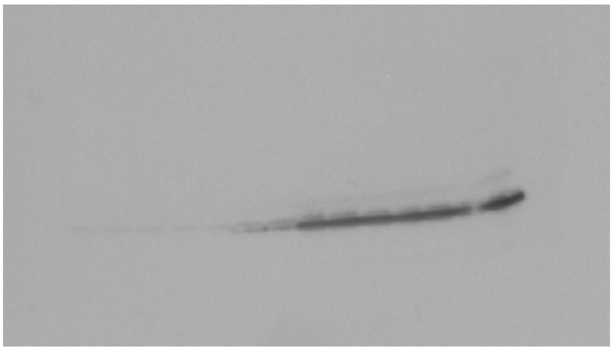

pY1062

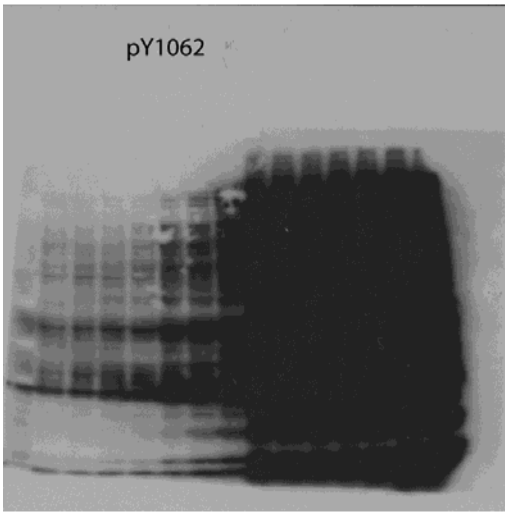

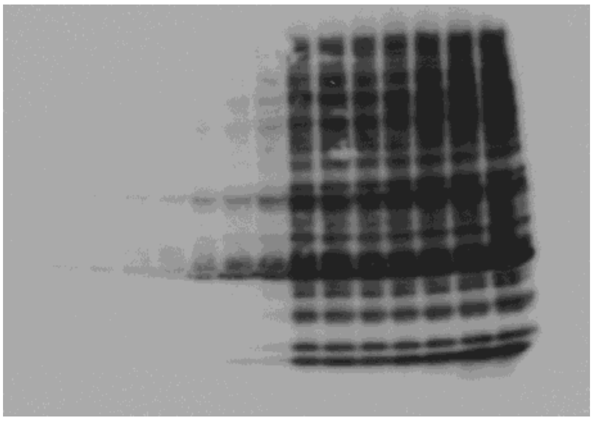

ponceau

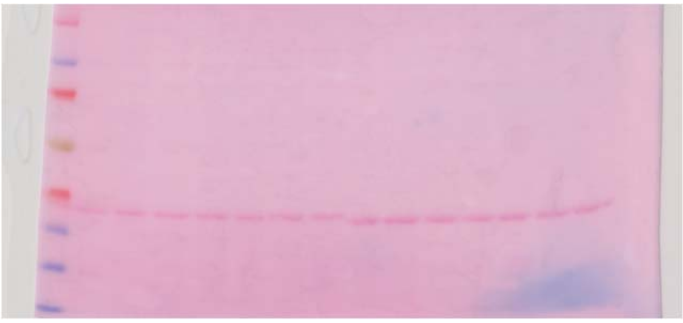

Supplementary Figure 9. Sources of immunoblotting data

Supplementary Table 1. Data collection and refinement statistics for RET S904F mutant bound to adenosine

**Data Collection**		
Spacegroup	$P4_32_12$	
Cell Dimensions (Å)	50.90, 50.90, 242.51	
α, β, γ (°)	90, 90, 90	
Resolution (Å)	48.5 - 2.08	(2.19 – 2.08)
R_{pim} (%)	4.6	(34.0)
Completeness (%)	98.5	(97.0)
Multiplicity	24.5	(25.5)
Wilson B	28.3	
CC(1/2)	99.8	(64.1)
Mean(I)/sd	12.8	(2.0)
**Refinement**		
Total No. reflections (free)	27003	(1364)
Resolution (Å)	2.3	
R_{work} / R_{free} (%)	19.8 / 23.7	
No. atoms		
Protein	2297	
Adenosine	19	
Formate/glycerol	9	
Waters	34	
B-factors		
Protein	51.3	
Ligands	53.3	
Rmsd		
Bond lengths (/Å)	0.005	
Bond angles (/°)	0.72	
Ramachandran		
Favoured (/%)	96.9	
Outliers (/%)	0.0	
Clashscore	2.6	
Rotamer Outliers	2.65	

Rmsd, root-mean-square deviation.

Parenthesis shows the value for the highest resolution shell.

Supplementary Table 2. Melting temperature (Tm) by thermal shift assay using purified RET kinase domain polypeptides

RET kinase domain	Tm derivative (°C)			
	No drug		Vandetanib	
	Non-phosphorylated (CIP treated)	Phosphorylated (ATP treated)	Non-phosphorylated (CIP treated)	Phosphorylated (ATP treated)
Wild-type	42.78 ± 0.27	43.00 ± 0.06	45.39 ± 0.11	49.11 ± 0.19
S904F mutant	44.28 ± 0.04	44.16 ± 0.04	46.94 ± 0.44	47.92 ± 0.17

Mean melting temperature (Tm) values with standard error.

Wild-type and S904F mutant proteins were either dephosphorylated using CIP-phosphatase or phosphorylated by the addition of Mg-ATP, followed by incubation with DMSO or 1 μM vandetanib.

Supplementary References

1. Mizukami, T., *et al.* Molecular mechanisms underlying oncogenic RET fusion in lung adenocarcinoma. *J Thorac Oncol* **9**, 622-630 (2014).
2. Manning, G., Whyte, D.B., Martinez, R., Hunter, T. & Sudarsanam, S. The protein kinase complement of the human genome. *Science* **298**, 1912-1934 (2002).
3. Tung, C.S., Harvey, S.C. & McCammon, J.A. Large-amplitude bending motions in phenylalanine transfer RNA. *Biopolymers* **23**, 2173-2193 (1984).
4. Weiner, S.J., *et al.* A new force field for molecular mechanical simulation of nucleic acids and proteins. *J. Am. Chem. Soc.* **106**, 765-784 (1984).
5. Corey, R.B. & Pauling, L. Molecular Models of Amino Acids, Peptides, and Proteins. *Review of Scientific Instruments* **24**, 621-627 (1953).
