# Supplementary material for: A secondary RET mutation in the activation loop conferring resistance to vandetanib
Source: Nat Commun. 2018 Feb 12;9:625. doi: 10.1038/s41467-018-02994-7 (PMC5809600; doi:10.1038/s41467-018-02994-7)
Supplement: Supplementary file 3 — Description of Additional Supplementary Files [file 41467_2018_2994_MOESM3_ESM.pdf]

Description of Additional Supplementary Files

File Name: Supplementary Movie 1
Description: Molecular dynamics (MD) simulation of wild-type RET kinase domain (KD) in complex with vandetanib. Movie representing a 1 µs MD simulation of the wild-type RET KD in complex with vandetanib is shown (see also Supplementary Figure 7f).

File Name: Supplementary Movie 2
Description: Molecular dynamics (MD) simulation of S904F mutant RET kinase domain (KD) in complex with vandetanib. Movie representing a 1 µs MD simulation of the S904F mutant RET KD in complex with vandetanib is shown (see also Supplementary Figure 7f).
